# Functional and Proteomic Analysis of *Streptococcus pyogenes* Virulence Upon Loss of Its Native Cas9 Nuclease

**DOI:** 10.3389/fmicb.2019.01967

**Published:** 2019-08-22

**Authors:** Nina J. Gao, Mahmoud M. Al-Bassam, Saugat Poudel, Jacob M. Wozniak, David J. Gonzalez, Joshua Olson, Karsten Zengler, Victor Nizet, J. Andrés Valderrama

**Affiliations:** ^1^Division of Host-Microbe Systems and Therapeutics, Department of Pediatrics, School of Medicine, University of California, San Diego, La Jolla, CA, United States; ^2^Skaggs School of Pharmacy and Pharmaceutical Sciences, University of California, San Diego, La Jolla, CA, United States; ^3^Department of Bioengineering, University of California, San Diego, La Jolla, CA, United States; ^4^Department of Pharmacology, School of Medicine, University of California, San Diego, La Jolla, CA, United States

**Keywords:** group A *Streptococcus*, *Streptococcus pyogenes*, CRISPR-Cas, Cas9, regulation, proteomics, bacterial virulence, pathogenesis

## Abstract

The public health impact of *Streptococcus pyogenes* (group A *Streptococcus*, GAS) as a top 10 cause of infection-related mortality in humans contrasts with its benefit to biotechnology as the main natural source of Cas9 nuclease, the key component of the revolutionary CRISPR-Cas9 gene editing platform. Despite widespread knowledge acquired in the last decade on the molecular mechanisms by which GAS Cas9 achieves precise DNA targeting, the functions of Cas9 in the biology and pathogenesis of its native organism remain unknown. In this study, we generated an isogenic serotype M1 GAS mutant deficient in Cas9 protein and compared its behavior and phenotypes to the wild-type parent strain. Absence of Cas9 was linked to reduced GAS epithelial cell adherence, reduced growth in human whole blood *ex vivo*, and attenuation of virulence in a murine necrotizing skin infection model. Virulence defects of the GAS Δ*cas9* strain were explored through quantitative proteomic analysis, revealing a significant reduction in the abundance of key GAS virulence determinants. Similarly, deletion of *cas9* affected the expression of several known virulence regulatory proteins, indicating that Cas9 impacts the global architecture of GAS gene regulation.

## Introduction

Clustered regularly interspaced short palindromic repeats (CRISPR) and CRISPR-associated (Cas) genes are recognized as an adaptive immune system that allows prokaryotic organisms to defend against plasmids, bacteriophages and transposons (Barrangou et al., 2007). CRISPR-Cas systems are widely distributed in many bacterial and archaeal genomes (Makarova et al., 2015; Burstein et al., 2016), and are evolutionarily classified in two main classes, with class II as the most representative and uniquely driven by the nuclease Cas9 (Makarova et al., 2015). Type II CRISPR-Cas systems occur only in bacteria, and not in archaea (Haft et al., 2005).

A variety of important human pathogens possess a type II CRISPR-Cas system, including bacterial species that cause acute or chronic infections (Louwen et al., 2014). Several lines of investigation support the notion that endogenous bacterial factors involved in stress responses and virulence gene regulation might interact to modulate the expression of CRISPR-Cas genes. For example, mutants in stress adaptation regulatory proteins RelAQ down-regulate *cas* genes in *Enterococcus faecalis* (Yan et al., 2009), deletion of the osmotic regulator OmpR represses *cas* gene expression in *Yersinia pestis* (Gao et al., 2011), and *Escherichia coli* two-component regulatory system (TCS) BaeSR modulates *cas* genes expression in response to cell envelope stress (Perez-Rodriguez et al., 2011).

Genomic analyses of virulence features in diverse pathogenic bacteria suggest roles of CRISPR-Cas beyond defense against foreign DNA and viruses, including potential involvement in regulation of endogenous gene expression (Mojica et al., 2005), including those encoding virulence factors (Kuenne et al., 2013). These hypotheses have been supported experimentally in a number of cases. For example, using Cas9 and tracrRNA as regulators, *Francisella novicida* represses a key surface-expressed lipoprotein (BLP), avoiding recognition of the pathogen by host cellular receptors (Sampson et al., 2013). In addition, CRISPR-Cas modulates swarming and biofilm formation in *Pseudomonas aeruginosa* (Zegans et al., 2009), CRISPR-associated Cas2 enhances intracellular infection by *Legionella pneumophila* (Gunderson and Cianciotto, 2013), a CRISPR type II system contributes to *Campylobacter jejuni* attachment to and invasion of human intestinal epithelium (Louwen et al., 2013), and *cas9* deletion reduces *Neisseria meningitidis* epithelial cell adherence and invasion (Sampson et al., 2013). Recently, inactivation of *cas9* in *Streptococcus agalactiae* was shown to impair epithelial cell adherence and macrophage intracellular survival, which is translated to decreased virulence of the Δ*cas9* mutant strain in zebrafish and murine infection models (Ma et al., 2018).

Although Cas9 nuclease is found in many bacterial genomes, the native source of the Cas9 used in genome engineering is *Streptococcus pyogenes* (group A *Streptococcus*, GAS). Dubbed the most significant genetic tool of the 21st century (Pennisi, 2013), GAS Cas9 enables precise and efficient gene editing in species ranging from bacteria (Jiang et al., 2013), to yeast (DiCarlo et al., 2013), to monkeys (Niu et al., 2014) and human cell lines (Cong et al., 2013). While the GAS CRISPR-Cas9 system is one of the best understood biochemically (Marraffini, 2016), its influence on the physiology and the pathogenesis of its native organism remain unknown. This is striking since GAS remains a top 10 cause of infection-associated mortality worldwide, producing a wide spectrum of diseases with multiple clinical manifestations, ranging from mild impetigo and pharyngitis, to severe invasive toxic shock syndrome and necrotizing fasciitis (Cunningham, 2000; Carapetis et al., 2005).

Group A *Streptococcus* possesses a multitude of surface-bound and secreted virulence factors that subvert innate defenses and allow the pathogen to survive and replicate in the human host (Walker et al., 2014; Valderrama and Nizet, 2018). Control of virulence gene expression in GAS involves a complex, interconnected network of TCS and specific and/or global transcriptional regulators. Together, these virulence regulators integrate environmental host cues with the pathogen’s own metabolic state, as well as feedback signals from the expressed genome, into a coordinated response (Vega et al., 2016).

In this study, we present evidence that endogenous Cas9 impacts GAS pathogenesis. Specifically, Cas9 is required for efficient GAS adherence to epithelial cells, growth in human blood, and full virulence in a murine skin infection model. Unbiased proteomic analysis shows how Cas9 influences the abundance of several key GAS virulence proteins and regulators of virulence gene expression.

## Results

### Generation of a GAS *cas9* Mutant (Δcas9)

**FIGURE 1:**
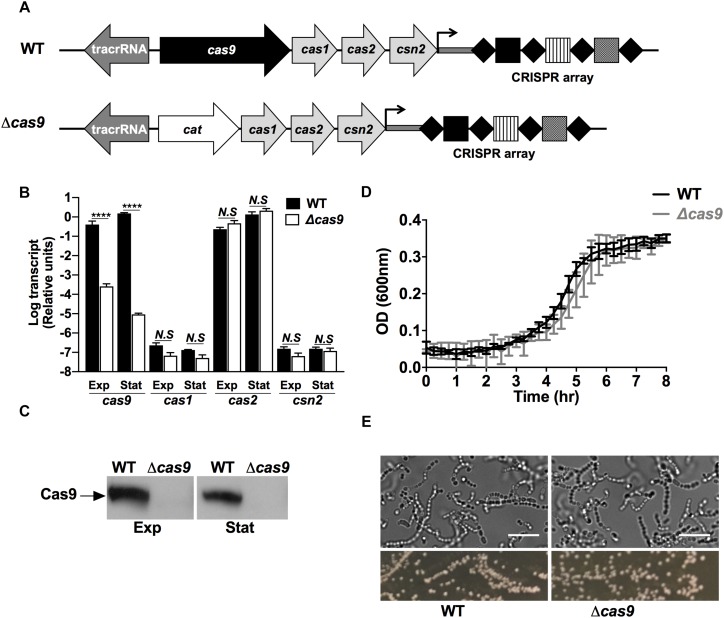
Deletion of the *cas9* gene does not affect GAS growth and morphology. **(A)** Schematic of the genomic organization of type II-A CRISPR-Cas loci in GAS 5448 wild type (top) and Δ*cas9* (bottom) strains. The *cas* genes are represented in light gray with *cas9* highlighted in black. The tracrRNA is shown in dark gray. Substitution of *cas9* by the *cat* gene in the Δ*cas9* strain is represented in white. The CRISPR array of GAS 5448 is constituted by the leader sequence (dark gray bar), four repeats (black diamonds) and three spacers (squares). **(B,C)** Quantification of *cas9*, *cas1*, *cas2*, and *csn2* mRNA transcripts by RT-qPCR **(B)** and expression of Cas9 protein by western blot **(C)** from wild type (WT) and *cas9* mutant (Δ*cas9*) GAS strains grown to mid-exponential (Exp) or stationary (Stat) cell growth phases. **(D)** Cellular growth curves of WT Δ*cas9* GAS strains grown at 37°C in THB media. **(E)** Microscopic visualization (top panels) and colonies morphology (bottom panels) of WT and Δ*cas9* GAS strains grown to stationary growth phase in THB media or on THA agar plates, respectively. Scale bar (10 μm) is indicated. For each experiment, samples were assayed at least in triplicate. Data in **(B)** and **(D)** are plotted as the mean ± SEM and are pooled and representative of three independent experiments, respectively. Data in **(B)** was analyzed by two-way ANOVA multiple comparisons. N.S, not significant (*p* > 0.05); ^****^P < 0.0001.

To explore the functional role of Cas9 in GAS, we generated a precise in-frame allelic exchange mutant in the background of the well characterized globally disseminated GAS serotype M1T1 strain 5448 (Chatellier et al., 2000), wherein the *cas9* gene in the type IIA CRISPR operon was replaced with chloramphenicol acetyltransferase (*cat*) gene (Figure 1A) as validated by PCR and sequence analysis. Functional confirmation of gene deletion in the GAS Δ*cas9* mutant strain was achieved in both mid-exponential and stationary growth phase cultures by real-time qPCR (Figure 1B) and western immunoblot using specific antibodies raised against the GAS nuclease (Figure 1C). Additionally, we confirmed that the replacement of *cas9* with the *cat* gene did not exert polar effects on the expression of downstream *cas* genes, since the transcriptional levels of *cas1*, *cas2*, and *csn2* did not differ significantly between WT and Δ*cas9* strains (Figure 1B).

Loss of Cas9 did not affect growth in bacteriological media (Figure 1D), and wild-type (WT) and Δ*cas9* GAS strains had similar morphology and chain length distribution in brightfield microscopy imaging (Figure 1E), and similar susceptibility to cell wall-active antibiotics vancomycin and penicillin (Supplementary Table S1). Genetic complementation of the Δ*cas9* strain with a plasmid expressing full-length *cas9* under a constitutive promoter restored production of the Cas9 protein product above WT levels (Supplementary Figure S1A); however, the complemented strain showed a significant defect in growth compared to both WT and Δ*cas9* strains (Supplementary Figure S1B). This result was consistent with Cas9 overexpression-mediated toxicity, limiting our attempts to complement specific Δ*cas9* phenotypes (Supplementary Figures S1C,D), while at the same time suggesting a non-canonical function of Cas9 in GAS.

### Cas9 Deficiency Is Associated With Reduced Abundance of Key GAS Virulence Determinants and Regulatory Factors

A number of studies in pathogenic bacteria have suggested Cas proteins play important roles in biological processes beyond the well-studied adaptive immune system that protects against foreign DNA (Mojica et al., 2005). To explore the overall functional impact of Cas9 on GAS pathophysiology, we compared the proteomic profiles of GAS WT and Δ*cas9* strains from cells grown to mid-logarithmic phase using quantitative, multiplexed proteomics (Lapek et al., 2018). We found that deletion of *cas9* resulted in drastic remodeling of the GAS proteome. From a total of 1,224 proteins identified, the abundance of 340 proteins was significantly decreased, and 405 proteins significantly increased, in the Δ*cas9* mutant compared to the WT parent GAS strain (Supplementary Figure S2 and Supplementary Table S2).

**FIGURE 2:**
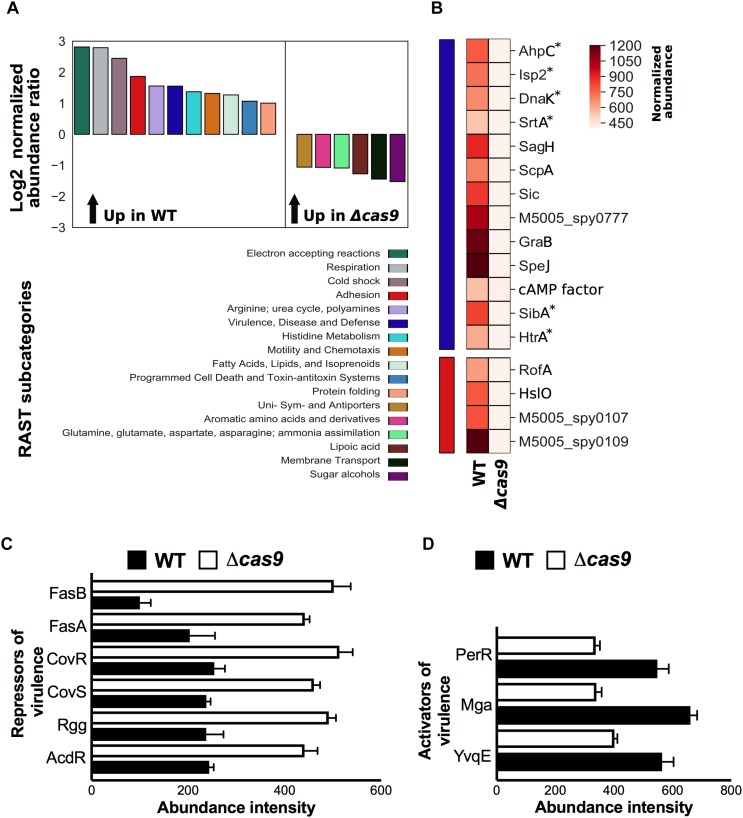
Cas9-deficiency is associated with RAST functional enrichment and differential abundance of GAS virulence determinants and regulators. **(A)** Comparison of significant differential protein abundance at the RAST subcategory level (Log2 normalized abundance ratio) between wild type (WT) and isogenic *cas9* mutant (Δ*cas9*) GAS strains. Significant enrichment in protein abundance in the wild-type (up in WT) or in the *cas9*-deficien mutant strain (up in Δ*cas9*) is indicated (top panel), with a matching color code of the corresponding RAST subcategory (bottom panel). **(B)** Statistical representation of the differential abundance of proteins identified in the WT or Δ*cas9* GAS strains and grouped under “virulence, disease, and defense” (blue bar) and “adhesion” (red bar) RAST subcategories. Color pattern of the normalized protein abundance is indicated **(C,D)** Comparison of normalized abundance of transcriptional repressors **(C)** and activators **(D)** of GAS virulence identified by proteomics in the WT and Δ*cas9* GAS strains. Proteins annotated with asterisks indicate that were manually added (curated) into the relevant RAST subcategory based on published evidence. All differential proteins listed in **(B,D)** were significant by *t*-test (*p* < 0.05).

To understand functional changes attributable to *cas9* deficiency, we undertook gene annotation by Rapid Annotations Subsystems using Technology (RAST server) (Aziz et al., 2008) and classified all identified proteins into subcategories by predicted gene function (Supplementary Table S3). Among the identified proteins, RAST analysis yielded 17 functional subcategories significantly affected by loss of Cas9, with 11 subcategories enriched in the WT strain, and 6 subcategories enriched in the Δ*cas9* mutant strain (Figure 2A). The most enhanced subcategories in the Δ*cas9* mutant strain were “Sugar alcohols” and “Membrane Transport” (Figure 2A), including glycerophosphoryl diester phosphodiesterase (encoded by M5005_Spy0647) and cofactor transporters (encoded by *cbiQ* and *cbiO1*) (Supplementary Table S3). In contrast, the most pronounced subcategories enriched in the WT strain were “Electron Accepting Reactions” and “Respiration” (Figure 2A), including important bacterial metabolic proteins such as arsenate reductase (encoded by *arcA*), glycerol dehydrogenase (encoded by *gdlA*), and ferrodoxin (encoded by M5005_Spy0616) (Supplementary Table S3). Together these observations suggest that loss of Cas9 may indirectly lead to imbalances in the metabolic status of GAS.

Of particular interest, virulence-related RAST subcategories were markedly reduced in the Δ*cas9* mutant compared to the WT GAS strain. To that end, we focused our proteomic analysis on the normalized abundance levels of individual gene products grouped within the “Adhesion” (red) and “Virulence, Disease, and Defense” (blue) RAST subcategories (Figure 2B). At least four proteins associated with GAS adhesion, including the chaperone Hslo, the transcriptional regulator of adhesins RofA, pilin (M5005_spy0109) and pilus ancillary protein 1 (M5005_spy01070) were significantly reduced in the Δ*cas9* strain, potentially impacting GAS host cell adherence and colonization. At least 13 additional GAS virulence-associated proteins had significantly reduced abundances in the Δ*cas9* strain (Figure 2), including immunogenic secreted products Isp2 and SibA, and proteins involved in folding and maturation of other secreted GAS virulence determinants. Among these is HtrA, a serine protease directly associated with maturation of two key pathogenic factors: cysteine protease SpeB and pore-forming toxin streptolysin S (SLS) (Lyon and Caparon, 2004).

Group A *Streptococcus* genes encoding proteins induced during human neutrophil phagocytosis (Voyich et al., 2003) were also significantly reduced in Cas9 deficiency, including detoxifiers of cell-damaging reactive oxygen species (ROS), such as AhpC and DnaK, and sortase A (SrtA), the transpeptidase required for cell wall anchoring of surface virulence factors such as M protein, GRAB and protein F (Barnett and Scott, 2002; Raz and Fischetti, 2008). Proteins involved in GAS evasion of the complement system, which has a central role in innate immunity, were also diminished in the Δ*cas9* strain, These included (a) serine protease ScpA that specifically cleaves C5a, a key chemoattractant factor that also helps coordinate activation of the classical, alternative and lectin-binding complement pathways (Cleary et al., 1992) and (b) *SIC* (streptococcal inhibitor of complement), which further inactivates antimicrobial factors including cathelicidin defense peptides, α-defensins, secretory leukocyte protease inhibitors and lysozyme (Cole et al., 2011).

Other virulence-related proteins found to be less abundant in the Δ*cas9* strain include protein G-related α2-macroglobulin-binding protein (GRAB), which protects important GAS virulence determinants from proteolytic degradation (Rasmussen et al., 1999); CAMP factor, linked with GAS epithelial cell adherence and resistance to macrophage phagocytosis (Kurosawa et al., 2016, 2018), SagH (an SLS export transmembrane protein), and superantigen SpeJ. The reduced abundance of virulence-related proteins was a first clue to the possibility of virulence attenuation in the Δ*cas9* mutant.

A complex network of two-component regulatory systems (TCS), global and specific transcriptional regulators exert efficient and rapid control over the expression of all the aforementioned GAS virulence-related proteins and other relevant pathogenicity factors. Since Cas9 controls key transcriptional regulatory elements in other pathogens (Ma et al., 2018), we next analyzed the differential abundance of virulence regulatory proteins between GAS WT and Δ*cas9* strains. Remarkably, the abundance of several transcriptional repressors of virulence were increased in the Δ*cas9* strain (Figure 2C). This included the master TCS CovR/CovS, which influences transcription of up to 15% of all GAS chromosomal genes, including repression of hyaluronic acid capsule, SLS precursor SagA, streptokinase (SkA), cysteine protease SpeB and other secreted GAS factors (Graham et al., 2002). Also increased in the Δ*cas9* mutant was the TCS FasA/FasB, which downregulates transcription of genes encoding GAS adhesins in a growth phase-dependent fashion (e.g., *fbp54*, *mrp*) (Kreikemeyer et al., 2001). Finally, transcriptional regulatory proteins Rgg, which downregulate several genes associated with GAS virulence (Chaussee et al., 2002) and AdcR, involved in the repression for adaptive responses to zinc limitation (Sanson et al., 2015) were also more abundant in the Cas9-deficient strain.

In contrast, transcriptional activators of virulence determinants were diminished in the Δ*cas9* strain (Figure 2D). One such example is Mga, the best-characterized stand-alone virulence regulator of GAS, which induces a core set of virulence genes, including M protein, the most abundant GAS surface protein. Similarly, protein abundance levels of the transcriptional regulator PerR and the histidine kinase YvqE were reduced in the absence of Cas9; these proteins are known to directly upregulate GAS responses to oxidative stress and thereby enhancing resistance and virulence in the host (Grifantini et al., 2011), and signaling-mediated control of biofilm formation and pilus expression (Isaka et al., 2016), respectively.

In summary, loss of Cas9 is associated with changes of several GAS virulence-related regulatory elements, generally fitting a pattern of reduced activators and enhanced repressors, suggesting an important role of the nuclease on the overall virulence of the bacterium.

### Loss of Cas9 Is Associated With GAS Virulence Attenuation

**FIGURE 3:**
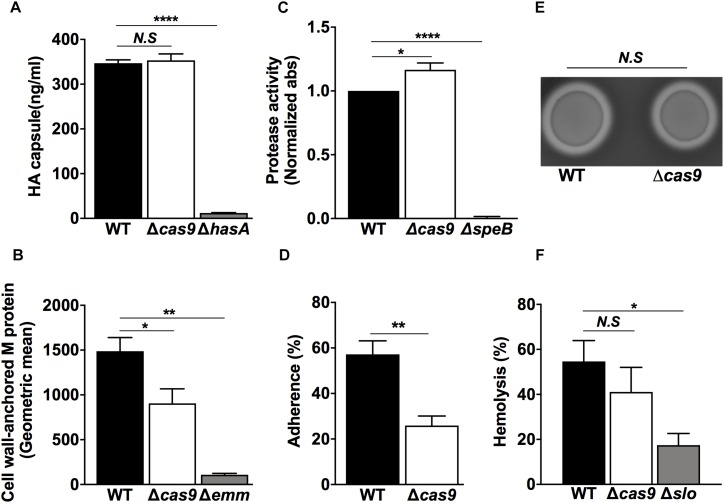
Lack of Cas9 is impaired with significant changes in key virulence factors and pathogenic functionalities of GAS. **(A–F)** GAS WT and Δ*cas9* strains were assessed for **(A)** Capsule expression by ELISA, **(B)** Quantification of M protein-anchored to the cell wall by flow cytometry, **(C)** SpeB protease activity by azocasein assay, **(D)** Capacity of adhesion to HaCaT human skin keratinocytes, **(E)** β-hemolysis on blood-agar media, and **(F)** Efficiency on human red-blood cell lysis. Isogenic GAS mutant strains in capsule (*hasA)*, M protein (Δ*emm*) SpeB (Δ*speB*), and SLO (Δ*slo*) were used as negative controls in **(A–C,F)**, respectively. For each experiment, samples were assayed at least in triplicate. Data in **(A–D,F)** are plotted as the mean ± SEM, pooled from three independent experiments and analyzed by Student’s *t* test. N.S, non-significant (*p* > 0.05); ^∗^*P* < 0.05; ^∗∗^*P* < 0.01; ^****^P < 0.001.

To functionally validate our proteomic observations pointing toward an altered virulence phenotype in the Δ*cas9* strain, we first compared the expression and/or activity of well-known GAS virulence determinants. The GAS hyaluronic acid (HA) capsule varies in thickness across different strains (Ashbaugh et al., 1998). High level HA capsule expression can produce a mucoid colony morphology and plays a critical role in resistance to opsonophagocytosis and evasion of the host innate immune response (Wessels et al., 1991; Dale et al., 1996). Visual comparisons between the WT and Δ*cas9* strains did not reveal differences in mucoid morphology of the bacterial colonies (Figure 1E, bottom panels), and the amount of capsular HA extracted from mid-exponential growth phase bacteria was similar in the two strains by hyaluronan specific ELISA (Figure 3A). These findings were consistent with the proteomics results showing similar expression of hyaluronan synthase (HasA) in both strains (Supplementary Table S2).

The surface-anchored M protein forms the basis for the serological differentiation of GAS strains, and influences several pathogenic properties of the bacterium such as epithelial cell adherence (Okada et al., 1995) and resistance to opsonophagocytosis. M protein can also bind several host components including fibrinogen and immunoglobulin G (Ghosh, 2018), and block membrane-lytic activities by sequestering antimicrobial peptides (LaRock et al., 2015) and histones (Dohrmann et al., 2017). M protein has pro-inflammatory properties that drive the pathogenesis of streptococcal sepsis (Herwald et al., 2004) and activate host IL-1β signaling through NLRP3 inflammasome activation (Valderrama et al., 2017). Using flow cytometry, we measured a significant reduction of cell wall-associated M protein in the Δ*cas9* strain compared to the WT strain (Figure 3B), consistent with a trend toward lower M protein detected by our proteomic experiments (Supplementary Table S2), though this fell short of statistical significance (*p* value = 0.06). Reduction of cell wall-associated M protein could be also attributed to the reduced amounts of the specific M protein transcriptional activator (Mga) and/or its surface anchor sortase (SrtA), found in the Δ*cas9* mutant by proteomics (Figures 2B,D, respectively).

M protein is also one of the multiple GAS virulence factors recognized and cleaved by cysteine protease SpeB, the most predominant secreted protein produced by the pathogen (Aziz et al., 2004; Nelson et al., 2011). SpeB contributes to the establishment of localized skin infections (Cole et al., 2006) and enhances GAS persistence and dissemination by degrading multiple host proteins (Eriksson and Norgren, 2003; Nyberg et al., 2004; Shelburne et al., 2005). Our whole cell proteomic analysis did not show differences in the abundance of SpeB between WT and Δ*cas9* strains (Supplementary Table S2) but did not capture the secreted protein fraction of SpeB. Thus, we directly studied extracellular protease activity of SpeB from bacterial supernatants of both strains using the azocasein assay (Hollands et al., 2008) and found a significant increase in protease activity in the Δ*cas9* strain vs. the WT counterpart (Figure 3C). This increased cysteine protease activity could contribute to the reduced surface-attached M protein observed in the Δ*cas9* strain (Figure 3B).

A primary step in GAS colonization of the host is adhesion to host epithelial cells (Nobbs et al., 2009; Brouwer et al., 2016). Adhesion-related proteins were highlighted in our proteomic analysis, with a significant reduction in the abundance of proteins, such as pilin and pilus components, in the Δ*cas9* mutant strain (Figures 2B,C). We compared adherence of WT vs. Δ*cas9* to human HaCaT keratinocytes and found a significant three-fold reduction in Δ*cas9* adhesion (Figure 3D), suggesting a Cas9-dependent effect on GAS host cell binding.

β-hemolysis is a hallmark phenotypic feature of GAS (Nizet, 2002) and the oxygen-stable streptolysin S (SLS) is the main factor responsible for red cell lysis on blood agar media. SLS forms hydrophilic pores in a broad array of epithelial and immune cell types (Miyoshi-Akiyama et al., 2005; Molloy et al., 2011). SagA, the secreted structural propeptide for the SLS toxin (Dale et al., 2002; Nizet, 2002) was not detected in our proteomic analysis, nor were there differences in abundance of the oxygen-labile pore-forming, cholesterol-dependent streptolysin O (SLO) another important secreted toxin (Madden et al., 2001), between the WT and cas9 strains (Supplementary Table S2). Consistent with these findings, the WT and the Δ*cas9* mutant strains did show significant differences in the zone of β-hemolysis surrounding the GAS colonies (Figure 3E) or hemolysis in a liquid phase red blood cell lysis assay (Figure 3F).

**FIGURE 4:**
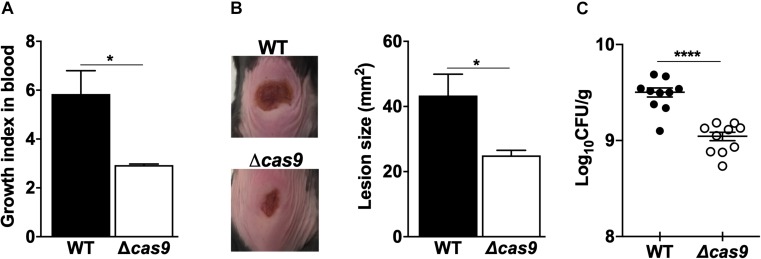
Loss of Cas9 attenuates GAS virulence. **(A)** Comparison of the ability of GAS WT and Δ*cas9* strains for growth on human whole blood. Growth index in blood was calculated as the ratio of recovered CFUs after incubation over the initial inoculum. **(B,C)** Subcutaneous infection of C57BL/6 mice with GAS WT and Δ*cas9* strains. **(B)** Representative images (left panel) of lesions triggered by GAS WT (top) or Δ*cas9* (bottom) strains, and average lesion sizes (right panel). **(C)** Enumeration of CFUs recovered from excised lesions 48 h post-infection. Data are plotted as the mean ± SEM and are pooled from three **(A)** and two **(B,C)** independent experiments and analyzed by Student’s *t* test. ^∗^*P* < 0.05; ^****^P < 0.001.

Together, our proteomic analysis and phenotypic assays suggested a Cas9-associated control over some of the key virulence determinants of GAS. To explore the cumulative effect of these changes, we first compared the capacity of WT vs. the Δ*cas9* GAS strains to proliferate in human whole blood *ex vivo* and found attenuated growth in the Δ*cas9* mutant (Figure 4A). Moving further to an *in vivo* infection model, we followed the development of necrotic skin ulcers following subcutaneous challenge of mice with WT vs. Δ*cas9* GAS strains. Twenty-four hours post-infection, lesions were significantly larger in WT GAS-infected mice compared to those challenged with the Δ*cas9* mutant (Figure 4B). When the mice were euthanized and lesions harvested for colony forming units (CFUs) enumeration 48 h after infection, a significantly higher amount of WT GAS bacteria were recovered compared to Δ*cas9* mutant bacteria (Figure 4C). In summary, these data suggest that Cas9 plays an important role during GAS infection *in vivo*, and this effect might reflect influences of the nuclease on several different virulence phenotypes and virulence-related regulatory factors.

## Discussion

The discovery and molecular characterization of RNA-programmable Cas9 nuclease emerged from basic research on the type II CRISPR-Cas system from GAS and has provided a revolutionary biotechnological tool for genome engineering, with promising potential to develop novel strategies to fight and cure many diseases (Le Rhun et al., 2019). Despite the attention that GAS Cas9 has received and the major health problem that GAS infections continue to exert on the public health, the native biological role of Cas9 and its contribution for GAS pathogenesis has yet to be reported. In this study, we provide initial experimental evidence that Cas9 has a significant effect on GAS virulence associated phenotypes *in vitro* and *in vivo*. These effects expand the biological significance of GAS Cas9 beyond its well-known role as the key component of the adaptive immune system that can precisely recognize and target foreign DNA.

Deletion of *cas9* in GAS did not affect growth kinetics nor gross morphology of the bacterium, consistent with observations upon loss of Cas9 orthologs in other organisms such as *F. novicidia*, *N. meningitidis* and GBS, where cell viability of Cas9 deficient strains was also not impacted (Sampson et al., 2013; Ma et al., 2018). To date, the evidence suggests that Cas9 is not involved in the control of essential gene products.

Cas9 RNA transcripts and protein levels were independent of GAS growth phase in bacteriological media, consistent with constitutive Cas9 expression; however, our experiments cannot exclude the possibility that changes in Cas9 expression occur *in vivo* during encounters of the pathogen with host factors. Our *in vitro* experiments demonstrate that *cas9* deletion is associated with a significant reduction in the GAS capacity to adhere to epithelial cells. Moreover, our *ex vivo* experiments in human whole blood infected with live WT or Cas9-deficient GAS bacterial strains show a significant contribution of endogenous Cas9 expression to bacterial growth, a key feature of the pathogen for dissemination within the host. During necrotic skin infection *in vivo*, absence of Cas9 was linked with diminished size of necrotic skin ulcers and reduced bacterial load within the harvested wounds.

Our proteomic studies suggest that the virulence phenotypes displayed upon loss of Cas9 are mediated in part by control over the abundance of at least four proteins related with GAS adhesion and other thirteen proteins associated with GAS virulence and defense, including pilus structural components, adhesins, key proteins that mediate resistance to reactive oxygen species, immunogenic secreted products, complement inhibitory factors and toxins. Additionally, our functional experiments show a reduction in M protein on the bacterial surface, which may be reflected by enhanced SpeB proteolytic activity in the Δ*cas9* mutant. Reduced SpeB activity is observed in the GAS transition to systemic infection (Aziz et al., 2004; Cole et al., 2011), and normal Cas9 function may be required for this functional shift.

Group A *Streptococcus* Cas9-mediated control of key virulence determinats finds a parallel in Cas9-mediated regulation of BLP in *F. novicida*, which enables the pathogen to dampen TLR2-dependent inflammatory response and to survive within host cells (Sampson et al., 2013). Also, the observed broad effect of Cas9 on GAS virulence regulation is reminiscent of the multiple virulence pathways regulated by the CRISPR-Cas9 system of *C. jejuni*, including those encoding lipoproteins, flagella, and chemotaxis-related factors (Shabbir et al., 2018).

**FIGURE 5:**
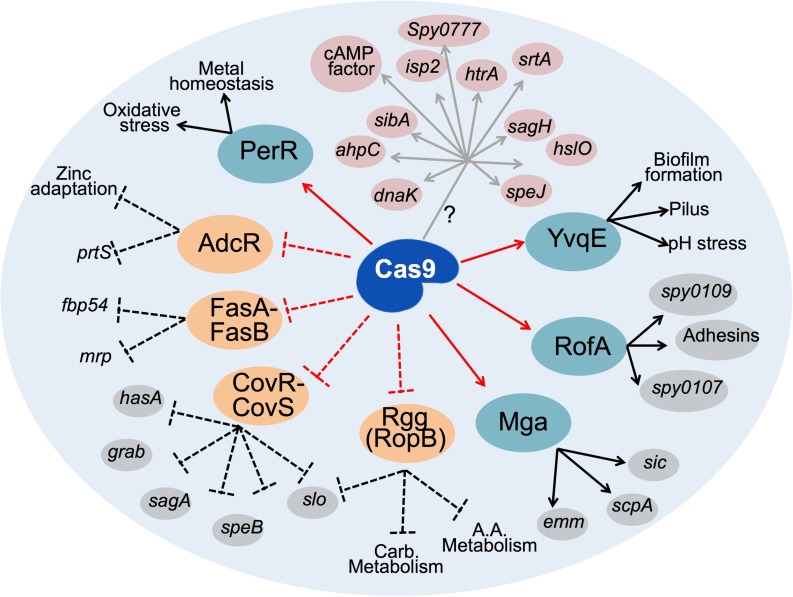
Schematic representation of the network of GAS regulatory proteins and virulence factors affected upon loss of Cas9. Cas9 may directly (gray arrows) activate expression of several GAS virulence determinants (highlighted in pink) through an unknown molecular mechanism (?). Cas9 also upregulates (solid red arrows) some transcriptional activators of virulence (highlighted in teal), further augmenting the expression of numerous virulence factors (black arrows). Conversely, Cas9 negatively controls (dashed red lines) the expression of several transcriptional repressors of virulence (highlighted in orange). Consequently, Cas9 blocks the repressor role (dashed black lines) of these regulators on the expression of key GAS virulence factors. Virulence determinants downstream of transcription factors experimentally confirmed in this study are highlighted in gray. Other virulence factors or functions known from previous studies to be regulated by the highlighted repressors and activators of GAS virulence are depicted with black text only. Carb. Metabolism, carbohydrates metabolism, A.A. metabolism, amino acids metabolism.

Absence of GAS Cas9 was associated with differential abundance in several virulence-related transcriptional regulatory factors, including enhanced levels of well-known transcriptional repressors of virulence. Conversely, abundance of important activators of virulence was diminished as a consequence of Cas9 deficiency, suggesting that Cas9 mediates a coordinated balance for the expression of the virulence machinery of GAS, including two of the most important and best studied GAS global regulators (e.g., the TCS CovR/CovS and the transcriptional regulator Mga). Similar evidence of Cas9 regulatory effect over virulence-related transcriptional regulators have been seen in GBS, where the nuclease influenced transcriptional regulator RegR, the modulator of hyaluronidase activity, a key virulence factor involved in GBS blood-brain barrier invasion during meningitis (Ma et al., 2018). Based on our experimental evidences, we present a schematic for Cas9-mediated virulence control (Figure 5), in which Cas9 could control the expression of several GAS virulence determinants, both directly or indirectly through its regulatory effect on the expression of key transcriptional regulators of virulence.

Since deletion of Cas9 is associated with significant changes in the abundance of more than 40% (*n* = 745) of GAS products encoded by genes dispersed throughout the genome, including those involved in diverse cellular processes such as stress response, protein metabolism, gene regulation and pathogenesis, among other functions, our studies suggest that Cas9 is a global regulator of GAS virulence and physiology.

Considering the highly specific endonuclease activity of Cas9, one potential mechanism underlying Cas9 effects on virulence regulation is that the nuclease may complex with the tracrRNA encoded inmediately downstram of Cas9 in the GAS genome (Figure 1A). In that manner, Cas9 could interact with operator regions of genes encoding the virulence determinants observed to be affected by loss of Cas9, leading to degradation or alteration of the corresponding transcripts. Further studies are required to address this hypothesis or other potential molecular mechanisms of Cas9-mediated regulation on GAS pathogenesis. Elucidating these mechanisms will help in understanding whether these new findings are associated with canonical functions of GAS CRISPR-Cas9 system, such as adaptive immunity mediated through spacer acquisition, and whether they can have direct impact on horizontal gene transfer with consequences on GAS evolution and ecology.

## Materials and Methods

### Bacterial Strains and Culture Conditions

GAS M1T1 5448 was originally isolated from a patient with necrotizing fasciitis and streptococcal toxic shock syndrome (Kansal et al., 2000). All GAS strains were routinely propagated at 37°C on Todd-Hewitt agar (THA, Difco) or in static liquid Todd-Hewitt broth (THB).

### Genetic Manipulation of GAS (Construction of Δ*cas9* Strain and Δ*cas9* Complementation)

Precise in-frame allelic replacement of the *cas9* gene was performed using established methodology (Pritzlaff et al., 2001). We first generated PCR products immediately up and downstream of the *cas9* gene. 1000 bp upstream was amplified with primers cas9upFw (5′-ccgctcgagtcctgtggagcttagtaggtttagc aagatggcagc-3′) and cas9upRv (5′-tatccagtgatttttttctccatttttgcctc ctaaaataaaaagtttaaattaaatcca-3′). Subsequently, 1060 bp of sequence downstream of *cas9* was amplified with primers cas9downFw (5′-tactgcgatgagtggcagggcggggcgtaatggctggttggcgt actgttgtggt-3′) and cas9downRv (5′-cccaagcttgacctgcattgattggat gctccaaatctcttgag-3′). Primers cas9upFw and cas9downRv were designed with 25 bp 5′ extensions corresponding to the 5′ and 3′ ends of the chloramphenicol acetyltransferase (*cat*) gene, respectively. The 660 bp fragment corresponding to the *cat* gene was PCR amplified from a previous GAS allelic replacement strain with primers catFw (5′-atggagaaaaaaatcactggatatacc-3′) and catRv (5′-ttacgccccgccctgccactcatcgca-3′). The upstream, downstream and full *cat* fragments were assembled in a second round of PCR using primers cas9upFw and cas9downRv. The resultant PCR amplicon was subcloned into temperature-sensitive vector pHY304, and allelic exchange mutagenesis in GAS 5448 was performed following double crossover as described previously to generate the stable mutant 5448Δ*cas9* strain. Attempt of Δ*cas9* complementation was carried out by amplification of *cas9* gene with primers cas9Fw (5′-tcccccc gggtgaaggaggcaaaaatggataagaaatactcaataggcttaga-3′) and cas9Rv (5′-gctctagatcagtcacctcctagctgactcaaatcaatgc-3′). The resulting PCR product was cloned into TOPO-XL vector (Invitrogen), subcloned into the multicopy plasmid pDCerm and transformed into the Δ*cas9* strain. Δ*cas9* strain complemented following the above mentioned protocol showed a significant defect in cell growth (Supplementary Figure S1).

### RNA Extraction and qPCR Assays

Total RNA was isolated from GAS bacteria pellets using RNEasy isolation kit (Qiagen), with an additional bead-beating step with 1.0 mm glass beads (Sigma). Synthesis of total cDNA was performed using iScript cDNA Synthesis kit (Bio-Rad). For each sample to be retrotranscribed, an exact amount of 1 μg of RNA was used as template. Real-time PCR assays were conducted in a CFX96 Real-Time System (Bio-Rad). Three biological and three technical replicates were analyzed for each sample. Reactions (20 μl) contained 1 μl cDNA, 10 μl SYBR fast qPCR Master Mix (KAPA biosystems) and 0.25 μM of each target-specific primer. Primer pairs cas9qPCRFw (5′-aaatacagaccgccacagtatc-3′) and cas9qPCRRv (5′-tcttccgacgtgtataccttcta-3′), cas1qPCRFw (5′-acgccaattggttgaa actc-3′) and cas1qPCRRv (5′-acgacggcatttagatacgc-3′), cas2q PCRFw (5′-ttgatatgccgacggacac-3′) and cas2qPCRRv (5′-aaaag cctccccaagaaatac-3′), csn2qPCRFw (5′-ggcggtacaattcttgtgct-3′) and csn2qPCRRv (5′-cgatttcacttcgggtttct-3′) and gyrAqPCR-Fw (5′-gaagtgatccctggacctga-3′) and gyrAqPCR-Rv (5′-cccgacctg tttgagttgtt-3′) were used to amplify transcripts from the *cas9, cas1, cas2, csn2*, and *gyrA* (encoding the DNA gyrase subunit A and used as an internal control to normalize the sample data) genes, respectively. Amplifications were carried out with 1 denaturation cycle (95°C for 5 min), followed by 45 cycles of amplification (95°C for 10 s; 60°C for 10 s; 72°C for 10 s). After amplification, melting curves were generated to confirm amplification of a single product. Relative *cas9* mRNA transcript levels were determined using ΔCt method and normalized with the mRNA transcript levels of *gyrA* housekeeping gene.

### Immunoblot Assays

Cells from GAS cultures were pelleted and lysed following enzymatic digestion with mutanolysin and lysozyme as previously described (Huang et al., 1989). Cell lysates were quantified for protein content by Pierce BCA Assay, and equal amounts of each sample analyzed were separated by SDS-PAGE, immunoblotted and visualized with Supersignal^®^ WestPico Chemiluminescent Substrate (Thermo Fisher Scientific) and CL-Xposure^TM^ Film (Thermo Fisher Scientific). The following antibodies were used for immunoblotting: anti-CRISPR-Cas9 (ab204448; abcam), ECL^TM^ anti-Rabbit IgG peroxidase-conjugated (NA934V; GE Healthcare).

### Proteomics Sample Preparation

Three independent cultures of mid-exponential growth phase GAS cells were pelleted and resuspended in lysis buffer containing 50 mM HEPES, 3% sodium dodecyl sulfate (SDS, Fisher), 75 mM NaCl (Sigma), 1 mM NaF (Sigma), 1 mM beta-glycerophosphate (Sigma), 1 mM sodium orthovanadate (Sigma), 10 mM sodium pyrophosphate (Sigma), 1 mM phenylmethylsulfonyl fluoride (PMSF, Sigma) and 1X cOmplete mini EDTA-free protease inhibitor cocktail tablet (Roche). Bacterial homogenates were sonicated to ensure complete lysis. Subsequent sample preparation, including 10-plex tandem mass tag (TMT) labeling, was performed as previously described (Lapek et al., 2018).

### Quantitative Proteomics, Protein Identification, and Analysis

Resulting “.raw” MS data files were processed using Proteome Discoverer 2.1 (Thermo Fisher). MS2 spectra were searched against a protein database derived from GAS strain MGAS5005 genome (GenBank: CP000017.2). Mass tolerances of 50 ppm and 0.6 Da were used for MS1 and MS2 spectra, respectively. Search parameters included full digest by trypsin with a maximum of two missed cleavages per peptide, static modifications of TMT 10-plex reagents on lysines and peptide N-termini (+ 229.162932 Da) and carbamidomethylation of cysteines (+ 57.02146 Da), and variable oxidation of methionine (+ 15.99492 Da). Results were filtered to a 1% false discovery rate using a target-decoy strategy at both the peptide and protein level.

For quantitative analysis, reporter ion intensities for the TMT reagents were extracted from MS3 spectra. Only peptide spectral matches exceeding an average signal:noise greater than 10 and an isolation interference less than 25% were retained for downstream analysis. Data were normalized as previously described (Lapek et al., 2017). Briefly, the reporter ion value for each peptide was summed to the protein level. The summed values were first normalized to the bridge channel value for each protein then to median of the entire bridge channel. To account for differences in peptide labeled, the quantitative information was then normalized to the median of the entire dataset and reported as the normalized, summed signal:noise ratios per protein, per sample. Datasets and corresponding annotated spectra are available through ProteomeXchange (PXD012568).

To determine significantly changing proteins, a *F*-test was first used to compare the variances of each protein in each condition. If the variances were equal, a standard Student’s *t*-test was performed, but if the variances were unequal, Welch’s correction was included. The GAS MGAS5005 genome was annotated in the RAST database in order to systematically organize genes into categories, subcategories, and subsystems. Differentially abundant proteins with *P* values < 0.05 were first identified. Normalized intensities for significant genes were summed per each RAST subcategory. Subcategories with > twofold change were plotted as the log2 of the WT/Δ*cas9* ratio (Log2 > 1 or < -1). Significantly changing proteins were also ranked using pi score as previously described (Xiao et al., 2014), considering significants all differential proteins with level alpha < 0.001. The “Virulence, disease and defense” and “Regulation of virulence” RAST subcategories were manually curated in order to ensure a comprehensive account of known virulence determinants and virulence regulatory proteins reported in the literature. All manually annotated proteins were highlighted as (^∗^) in Figures 2B,D and Supplementary Table S2. Python 2.7 was used to plot and analyze. The data and relevant code are available upon request.

### Hyaluronic Acid Capsule Assays

Hyaluronic acid extraction was performed as previously described (Hollands et al., 2010). Briefly, pellets from 5 mL GAS cells grown to mid-logarithmic were resuspended in 500 μl deionized water. In a 2 mL screw-cap tube, 400 ul of cells suspension and 1mL chloroform (Sigma-Aldrich) were combined and vortexed on max speed for 10 min. Samples were then centrifuged at 13,000 × *g* for 10min. The resulting aqueous phase was collected and diluted 1:40 for Quantikine hyaluronan ELISA (DHYAL0; R&D Systems) for quantification according to manufacturer’s instructions.

### Quantification of Cell Wall-Attached M Protein by Flow Cytometry

Mid-exponential phase GAS cells were probed for surface-attached M protein as previously described (van Sorge et al., 2014). Briefly, bacteria pellets were washed with PBS before incubation with mouse anti-serum raised against M1 protein. Samples were subjected to additional PBS wash steps to remove excess of antibodies, following incubation with goat anti-mouse IgG (H + L) AlexaFluor 488 secondary antibodies (Thermo Scientific). Samples were run through FACS Canto II (BD) without fixation and analyzed on FlowJo X7 (TreeStar).

### Cysteine Protease Activity Assays

SpeB protease activity was determined as previously described (Collin and Olsen, 2000). Briefly, overnight GAS cultures were diluted 1:50 into fresh THB media and cultured for 17 h at 37°C to early stationary phase. Cultures were centrifuged at 3,200 × *g*, and supernatants were filter sterilized. Equal volumes of filtered supernatant and activation buffer (1 mM EDTA, 100 mM sodium acetate, and 20 mM freshly prepared DTT) were mixed and incubated at 40°C for 30 min. 2% azocasein (Sigma) was dissolved in activation buffer and added to the activated supernatant in a 1:1 (v/v) ratio. The mixture was then incubated 1 additional h at 40°C. Excess azocasein was precipitated with the addition of trichloroacetic acid (Sigma) to a final concentration of 15% (w/v) and centrifuged for removal. Supernatants were transferred into a 96-well plate. Absorbance was measured at 366nm and normalized to wild-type levels to determine relative protease activity.

### Hemolysis Assays

Red blood cells (RBC) were prepared for hemolysis from whole blood drawn from healthy volunteer donors. Hirudin tubes-containing whole blood were left to settle naturally at room temperature on the bench for 1 h before washing the RBC pellet with PBS. The fraction of RBC was resuspended in PBS to 2% (v/v). Equal volumes of mid-logarithmic growth phase bacteria and 2% RBC suspension were mixed in V-bottom 96 well plates. After incubation for 1 h at 37°C, plates were spun down to pellet intact RBC. Supernatant was transferred to a new plate for OD measure at 450 nm. Percent of lysis was calculated using PBS as a negative control (0% lysis) and 0.025% Triton X100 as a positive control (100% lysis). For blood agar hemolysis, 10 μl of mid-logarithmic phase bacterial cultures were spotted on to 5% sheep blood in tryptic soy agar base (Hardy Diagnostics A10). Plates were incubated overnight at 37°C, imaged with ruler using Gel Doc XR + gel documentation system (Bio-Rad) and resulting zones of hemolysis were quantified for average radius using FIJI (Schindelin et al., 2012).

### Whole Blood Assays

1 × 10^6^ colony forming units (CFU) from mid-exponential growth phase GAS were resuspended in 20 μl PBS and mixed with 80 μl of whole blood. Samples were incubated at 37°C with rotation. After 2 h of incubation, 10 μl aliquots were diluted, plated on THA plates and incubated overnight for CFU enumeration. Blood was drawn from healthy volunteer donors into hirudin tubes by trained phlebotomists following a protocol for simple phlebotomy approved by the UCSD IRB/Human Research Protection Program. All subjects gave written informed consent in accordance with the Declaration of Helsinki.

### Adherence Assays

Adherence assays were performed as described previously (Timmer et al., 2006) but using the HaCaT human skin keratinocyte cell line. HaCaT cells were obtained from ATCC and propagated as monolayer in RPMI 1640 medium + 10% fetal bovine serum (FBS). For assays, cells were plated at 5 × 10^5^ cells/well in 24 well plates. Immediately prior to assay, the culture media on the HaCaT cells was replaced with fresh RPMI 1640 + 2% FBS. GAS strains grown to the mid-exponential phase were resuspended in RPMI 1640 + 2% FBS and added to the HaCaT cells at a multiplicity of infection (MOI) of 10. Plates were centrifuged at 800 g × 5 min to ensure GAS-HaCaT contact. Infected cells were incubated at 37°C with 5% CO_2_ for 30 min then lysed with trypsin and 0.025% Triton X100, serially diluted, and plated onto THA plates for CFU enumeration.

### Animal Experiments

The UCSD Institutional Animal Care and Use Committee approved all animal use and procedures. In compliance with ethical guidelines, to minimize the number of animals, we used a minimum of five mice for each experimental group (except where indicated in the figure legends) to ensure statistical power. All mice were randomly distributed into the different groups as indicated in the corresponding figure legend. 8- to 10-week-old C57BL/6 mice were infected subcutaneously with 1 × 10^8^ CFUs of either GAS wild type or Δ*cas9* strains resuspended in 100 μl of PBS. Lesions were imaged daily and surface area quantified using ImageJ software. At 48 h post-infection, lesions were excised, homogenized, and plated as dilutions onto THA plates for enumeration of bacterial CFU.

### Statistical Analysis

The data were collected from three independent experiments in triplicate, unless otherwise indicated. Data were combined and represented as mean ± SEM. Results were either analyzed by unpaired Student’s *t*-test or by two-way ANOVA using GraphPad Prism version 7. *P* < 0.05 was considered statistically significant.

## Data Availability

The raw data supporting the conclusions of this manuscript will be made available by the authors, without undue reservation, to any qualified researcher.

## Ethics Statement

Animal Subjects: The animal study was reviewed and approved by the UCSD Institutional Animal Care and Use Committee approved all animal use and procedures.

## Author Contributions

JV and VN developed the concept of the project. NG, VN, and JV wrote the manuscript and designed the experiments. NG, SP, JW, JO, and JV performed the experiments. NG, MA-B, JW, VN, and JV analyzed and interpreted the results. DG and KZ provided inputs to the execution of the project.

## Conflict of Interest Statement

The authors declare that the research was conducted in the absence of any commercial or financial relationships that could be construed as a potential conflict of interest.
