# Supplementary material for: Functional and Proteomic Analysis of Streptococcus pyogenes Virulence Upon Loss of Its Native Cas9 Nuclease
Source: Front Microbiol. 2019 Aug 22;10:1967. doi: 10.3389/fmicb.2019.01967 (PMC6714885; doi:10.3389/fmicb.2019.01967)
Supplement: Supplementary file 3 [file Data_Sheet_1.docx]

Supplementary Material

**Supplementary Figures**

**Supplementary Figure 1.** **Genetic complementation of Δ*cas9* GAS M1T1** **5448 strain leads to marked growth defect.** (**A**) Western blot analysis of Cas9 protein expression in wild type (WT), cas9-deficient (∆*cas*9) and *cas9*-plasmid complemented (pCas9) GAS strains from cells grown at stationary or exponential growth phases. (**B**) Cellular growth curves of WT, ∆*cas*9 and pCas9 GAS strains in THB media at 37°C. GAS strains in (**B**) carried out either the empty plasmid control (pDCerm) or the *cas9* expressing plasmid (pDCerm-*cas9*). (**C and D**) Subcutaneous infection of C57BL/6 mice with GAS WT, *∆cas9* or Cas9-complemented (pCas9) strains. Average lesion sizes (**C**) and enumeration of CFUs recovered from excised lesions (**D**) 48 h post-infection. Data in **C** and **D** are plotted as the mean ± s.e.m and are pooled from two independent experiments and analyzed by Student’s t test. N.S, (non significant) (p>0.05); ****P<0.001.

**
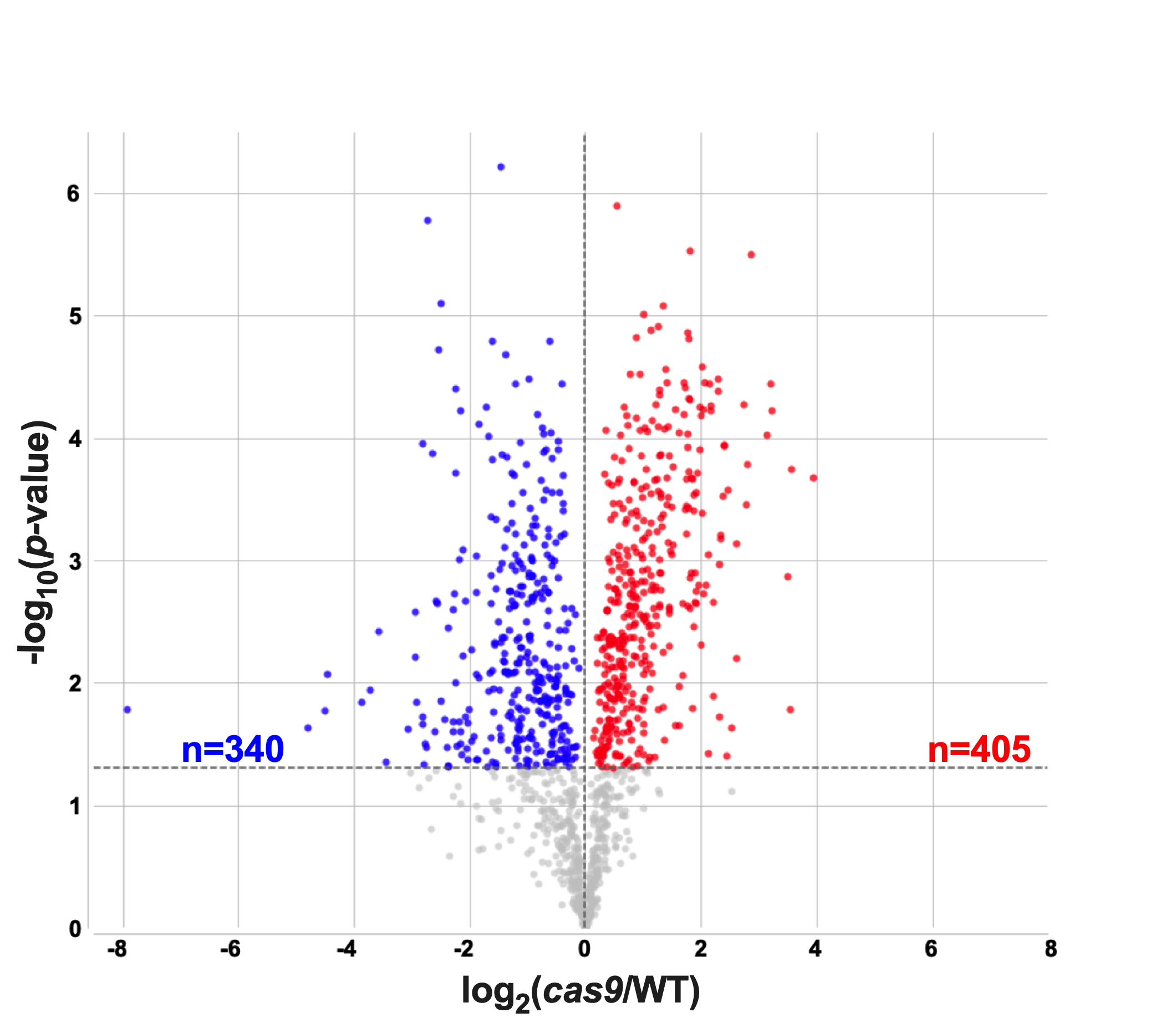
**

**Supplementary Figure 2.** **Abundance of proteins detected by tandem mass tag proteomics in WT** **and** **∆*cas9* GAS M1T1 5448 strains.** Volcano plot shows proteins significantly more abundant in the WT (blue dots, n=340) or in the ∆*cas9* strains (red dots, n=405). Significant proteins were initially identified by Student’s t test with *p* < 0.05.

**Supplementary Tables**

**Supplementary Table 1. Minimal Inhibitory Concentration (MIC) of cell wall synthesis inhibitory antibiotics against WT or ∆*cas*9 GAS M1T1 5448 strains** ^†^.

| Antibiotic | WT | Δ*cas9* |
| --- | --- | --- |
| penicillin G, μg/ml | 0.015-0.031 | 0.015-0.031 |
| vancomycin, μg/ml | 1.0-2.0 | 1.0-2.0 |

† MIC performed in Dulbecco’s Modified Eagle’s Medium (DMEM) supplemented with 10% Todd-Hewitt broth

**Supplementary Table 2. TMTproteomics.xlsx Normalized abundance of all proteins detected from WT and ∆*cas9* GAS 5448 strains by tandem mass tag proteomics**. Peptide reads were searched against database derived from the MGAS5005 M1 serotype *S. pyogenes* genome (GenBank: CP000017.2). Columns include: GenBank protein ID, protein description and full-raw data from three independent biological samples corresponding to each protein identified. F test, *P* value and pi score statistical analysis are also listed in the corresponding column. #, p<0.05 (proteins statistically significant by Student’s t test). *, level alpha<0.05 (proteins statistically significant by pi score).

**Supplementary Table 3. RAST_significant.xlsx Normalized protein abundance of proteins that were identified to be significantly enriched either in WT or in ∆*cas9* GAS 5448 strains**. Significant proteins were grouped in subcategories using RAST. Out of the 745 significantly differentially quantified proteins between both strains, 474 proteins were found annotated by RAST server (RAST annotation +). Some proteins annotated by RAST in different categories (RAST annotation +/-) or not annotated (RAST annotation -) were manually added into the corresponding functional category base on reported literature. Columns include: GenBank protein ID, corresponding gene name, protein description, RAST category and subcategory, as well as full-raw data from three independent biological samples corresponding to each protein identified as significant. F test, *P* value and pi score statistical analysis are also listed in the corresponding column. #, p<0.05 (proteins statistically significant by Student’s t test). *, level alpha<0.05 (proteins statistically significant by pi score).
